# Impact of Agricultural Division of Labor on Fertilizer Reduction Application: Evidence from Western China

**DOI:** 10.3390/ijerph20053787

**Published:** 2023-02-21

**Authors:** Shiyao Zhou, Chen Qing, Jia He, Dingde Xu

**Affiliations:** 1 College of Management, Sichuan Agricultural University, Chengdu 611130, China; 2 Sichuan Center for Rural Development Research, College of Management, Sichuan Agricultural University, Chengdu 611130, China

**Keywords:** horizontal division of labor, vertical division of labor, specialization in production, socialized services, fertilizer reduction, China

## Abstract

Nowadays, environmental friendly agriculture has become the world trend of modern agricultural development; fertilizer reduction application is an essential way to achieve sustainable development goals. With the deepening development of the agricultural specialized division of labor and socialized services, the division of labor economy can promote the economic input of fertilizer. Based on 540 survey data of farmers in the main rice-producing areas of Sichuan Province, this paper constructs a theoretical analytical framework for the impact of agricultural division of labor on fertilizer reduction application. A binary probit model was used to empirically analyze the effect of agricultural division of labor on fertilizer reduction application, and its mechanism was examined. The results show that: (1) both horizontal and vertical agricultural divisions of labor have positive and significant effects on the reduction in fertilizer application by rice farmers. All above results remain stable after treatment of endogeneity; (2) due to the migration of the rural labor, the horizontal division of labor is expressed as changes in the structure of labor and cultivation within the household which has changed. To achieve economies of scale, farmers increase specialization in production, resulting in reducing marginal cost and application of fertilizer; (3) the vertical division of labor is expressed in the adoption of external socialized services by farmers, which improves the land resource endowment of fragmentation and hydraulic conditions. Thus, it creates a good environment for fertilizer application to improve its application efficiency, which in turn promotes fertilizer reduction by farmers. Based on this, this paper proposes that the government should motivate farmers to deepen their participation in the horizontal and vertical division of labor. Meanwhile, it is also necessary to improve the agricultural specialization continuously and further promote the development of socialized services market.

## 1. Introduction

Fertilizer is one of the most important production elements in agricultural production activities [1]; the appropriate amount and scientific input have a significant impact on improving the yield of crops [2,3]. China has fed 22% of the world’s population on 7% of the world’s cultivated land, which is due to the application of chemical fertilizers. Fertilizer contributes to more than 40% of China’s grain yield increases [4,5], which has made a considerable effort to solve the problem of world food security. In 2021, fertilizer use and management have been added to the 25 core indicators for measuring the Sustainable Development Goals (SDGs) on food and agriculture-related issues [6]. However, excessive and inefficient application of chemical fertilizers is widespread in China [7], which causes problems, such as land degradation and non-point source environmental pollution [8]. The average fertilizer use in China was 21.9 kg per mu, far exceeding the world average of 8 kg per mu in 2015, while the effective utilization rate of organic fertilizer was less than 40 percent [9]. Under this background, the Chinese government has paid great attention to the problem of excessive and inefficient application of chemical fertilizers [10]. In 2015, the Ministry of Agriculture published the issuance of “to 2020 zero growth in fertilizer use action plan” and “to 2020 zero growth in pesticide use action plan”. From 2016 to 2021, the “Central Documents NO.1” has emphasized the importance of reducing chemical fertilizers and increasing efficiency to promote sustainable agricultural development for six consecutive years [11]. These policies have been partially effective in promoting chemical fertilizer reduction [12]. However, the promotion of chemical fertilizer reduction growth rate is low and still has challenges. According to statistics, the utilization rate of chemical fertilizers for China’s three major grain crops reached 40.2%, only 5 percent higher than in 2015 by the end of 2020 [13]. Fertilizer reduction application has become an essential task to promote the green and high-quality development of agriculture and still has a long way to go.

As the main subject of fertilizer reduction in agricultural production activities, the fertilizer application behavior of farmers and motivating mechanism have been a heated topic of discussion in academics and politics. However, academic research on fertilizer reduction by farmers has mainly focused on the following three aspects from the available studies. First, it focuses on the farmers themselves, mainly on the impact of individual or household characteristics in the reduction in their fertilizer application. For example, Yang et al. [14] used data from a survey of farmers in Hubei province and found that social capital had a significant positive impact on the increase in organic fertilizer application by farmers; Qiao and Huang [15] used data from China Cotton farmers Survey, finding that risk-averse farmers used more fertilizer than risk-taking farmers when the elasticity of fertilizer application and desired effects were high. Secondly, it focuses on the object of production, mainly on the impact of land as a production vector on fertilizer reduction. For example, Lu and Xie [16] used data from a survey in Jiangsu province and found that transfers of land use had a positive effect on fertilizer reduction; based on data from a survey of wheat farmers in northern China, Wu et al. [17] found that land scale management had a positive effect on fertilizer reduction. Third, it focuses on the impact of the external environment or policy on fertilizer reduction. For example, Guo et al. [18] used data from a household survey by the Rural Economy Research Center of the Ministry of Agriculture of China from 2014 to 2018 and found that agricultural subsidies had a significant negative effect on the use of fertilizer and contribute to fertilizer reduction. Based on CRHPS Data, Zhu et al. [19] found that agricultural machinery socialized service facilitated the adoption of fertilizer reduction technologies by farmers.

The above research established the foundation for this study. However, we note that, although a number of studies have been conducted on the impact of fertilizer reduction on farmers, few studies have taken the focus on the impact of the agricultural division of labor on fertilizer reduction on farmers from the perspective of the agricultural division of labor. The division of labor theory originates from Adam Smith’s *The Wealth of Nations*, which confirmed the importance of division of labor and specialization for economic growth. In classical economics, the essential of scale economies is in the division of labor and specialization, embedded in the economy of the division of labor [20]. Smith [21] indicated that the agricultural field has natural and endogenous barriers to the division of labor, owing to the characteristics of agriculture itself. Schultz’s hypothesis suggested that agricultural change in old factors or input of new factors is external. When it comes to neoclassical economics, Marshall [22] explored economies of scale, increasing return to scale and organization of the effects on the division of labor in the book *Principles of Economics*. Young [23] further pointed out that the degree of division of labor is the determining factor of economic growth. Nevertheless, the traditional divisions of labor theories mentioned above are limited to the industrial sector, and the study of the agricultural division of labor is extremely limited. According to the mainstream ideology, the seasonality, cyclicality, and non-divestiture of agriculture limit the division of labor and generate higher costs when compared to industry. In this way, agriculture can only be involved in industrial mechanisms, increasing the level of specialization and obtaining the economy of the agricultural division of labor in a roundabout way [24]. Yet, some scholars argued that this explanation of the division of labor in agriculture is incomplete, overemphasizing the special characteristics of agriculture and generally ignoring the division of labor in the agricultural field [25]. The traditional theory had ignored the contributions of the division of labor transactions, such as technical progress, labor substitution, and cost alternatives to the efficiency of production under an open market. It is irrelevant to the modern agricultural current development [26]. With the involvement of small farmers in the division of labor economy, it has brought efficiency much more than the one of tradition, and the agricultural division of labor has become an essential origin and driving factor of agricultural growth [27]. However, some scholars take the opposite attitude. They argued that the division of labor in agriculture, though leading to a short-term economic and efficient increase, at the same time resulted in specialized, homogeneous, and scale agriculture, thus destroying the environment. It is the essential requirement of sustainable and ecological agricultural development to reverse the division of labor [28]. Overall, most studies on the impact of the agricultural division of labor in fertilizer reduction are focused on the theoretical aspects [29]. However, few empirical studies have focused on or answered whether the agricultural division of labor has an impact on fertilizer reduction by farmers. If so, how much of an impact does it have? This is the critical question to be answered in this paper.

Compared to existing studies, this paper has the following marginal contributions: (1) under the background of prosperous development of agricultural specialization and socialized services, the economy of scale that comes from the division of agricultural labor has become an essential source of agricultural growth. It has been well confirmed in practice and empirical evidence. Therefore, this paper assumes that promoting increased rice yields while reducing chemical fertilizer use and the agricultural division of labor may facilitate the fertilizer reduction behavior of farmers. With that, this paper innovatively establishes a theory analysis framework of the impact of the agricultural division of labor on fertilizer reduction application. This theory analysis framework can explain how rice farmers are supposed to adopt optimal fertilizer application behavior under the background of the massive migration of rural labor and expanding market of agricultural machinery. (2) Within the theory analysis framework guidance, this study empirically analyzes the impact of agricultural horizontal division of labor and agricultural vertical division of labor on fertilizer application by rice farmers and their specific mechanisms of action. Additionally, they have made a series of robustness tests and heterogeneity analyses. The conclusions of this study can be used as a reference for policies regarding agricultural specialization, socialized services, and fertilizer reduction in rural areas.

## 2. Theoretical Analysis

The profit maximization theory of the small farmers’ school, which is represented by Theodore Schultz, believes that when farmers make resource decisions and element inputs, they use the resources, costs, and benefits available to make the best choice after weighing. For a long time, fertilizer and other elements inputs have been an increasingly expensive part of agricultural production [30], which accounts for 20–30% or more of the total cost of cultivation [31]. Some studies have shown that the cost of fertilizer application, regardless of the external environmental costs, has already exceeded its output benefits in economics alone [32]. Farmers, as economic men, pursuing profit maximization and risk minimization, will keep increasing fertilizer inputs to reduce economic losses [33], but it will continuously cause excessive use of fertilizers that do not maximize the economy. It will result in a long-term vicious circle. Thus, how to utilize efficiently and how to reduce the application to achieve economy of scale becomes an urgent task to ensure food production.

The increasing return to scale from the division of labor, on one hand, is the deepening of specialization of the farmers as the subject of management. On the other hand, production efficiency is improved through coordination and cooperation among different individuals or industries and by lengthening the roundabout production chain [34]. The division of labor can be expressed in two aspects: horizontal specialization and vertical socialized services. Among these, the horizontal division of labor is the specialization of cultivation based on natural endowment conditions. The vertical division of labor is the specialization of production and division of labor, which is formed in the process of producing due to the different efficiency among different production subjects and inputs, which is shown as the out-sourcing of socialized services [27]. Specialization and socialized services have become important expressions of the deepening agricultural division of labor [35]. The agricultural division of labor will lead to increases in productivity, reduction in production costs, and technological and organizational innovations [36].

For the agricultural horizontal division of labor, the capacity to use labor resources is used to measure the degree of specialization of the horizontal division of labor [37]. Horizontal division of labor in agriculture refers to agricultural specialization formed by farmers by expanding part of their cultivation scale [26], which is manifested in the degree of specialization in cultivation of contiguous areas of rice [26]. Specialization can provide an important impetus to labor resources and reduced fertilizer application by farmers [38]. On the one hand, with the migration of rural labor, Chinese farmers’ agricultural production has evolved from traditional farming dominated by labor to dependence on chemical agricultural resources such as fertilizers [39]. Farmers tend to reduce the fertilizer application frequencies and increase the amount of fertilizer per application to compensate for the negative effects of labor shortage [40]; on the other hand, farmers adjust their production according to the price of relative input elements, and, similarly, they tend to choose less labor but more chemical fertilizer [41]. At the same time, as the agricultural division of labor deepens, the lower cost of input intermediate inputs also continuously rejects traditional labor [42]. Increasing non-farm incomes also reduce the financial limits on agricultural production, which enables farmers to afford to use more fertilizer [43]; the structure of agricultural income reflects household part-time employment, which influences the level of specialization in agricultural production and therefore the division of labor [44]. Farmers who have a higher degree of part-time employees have a higher risk aversion and will input more fertilizer for short-term benefits [37,45], andthis leads to production shifts from traditional grain crops to more profitable commercial crops that require more fertilizer [46]. All these above adjustments are made by farmers to achieve scale economies. Thus, rational adjustment of the structure would help to decrease the marginal input cost and facilitate the fertilizer reduction behavior of farmers [16]. Therefore, it is necessary to adjust the internal elements input structure and adopt machinery instead of manual labor in order to improve the specialization degree, thus leading to a decrease in the original elements inputs, obtaining economies of scale, and eventually achieving fertilizer reduction.

For agricultural vertical division of labor, some scholars found that, under the current opening market of production elements for services, the involving socialized division of labor and productive outsourcing service can generate economies of scale [35]. Specifically, the division of labor in agriculture is mainly limited by transaction cost restraints, and intermediate socialized service organizations can save these costs [41]. The division of labor in various stages of the agricultural production process has not only improved production efficiency, but also reduced operating costs through the adoption of both manual and machine or total mechanical service production methods [27]. As for the growth of modern elements, such as technology and machinery for land inputs and the substitute of new elements for traditional ones, these achieve an increase in agricultural efficiency [47]. The improvements in land size, fertility, hydraulic conditions, and other characteristics can obviously increase the degree of agricultural vertical division of labor and also affect the fertilizer use of farmers [19]. While social services have increased the degree of the agricultural division of labor, the deepening of the agricultural division of labor has, in turn, given rise to the development of rice services. With the migration of rural non-farm employment, the number of surplus rural laborers decreases, showing aging, which contributes to the development of socialized services and thus further increases the degree of the agricultural division of labor [48]. Therefore, for the traditional land characteristics improvement, new elements need to be substituted and innovated from the outside to improve the production efficiency while promoting the reduction in fertilizer application.

**Figure 1:**
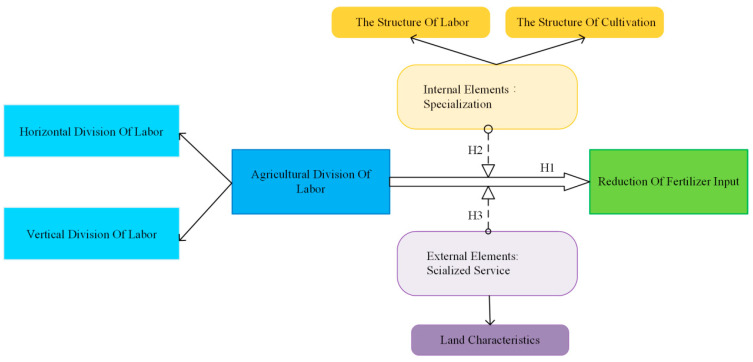
Mechanism for the impact of agricultural division of labor on fertilizer reduction application.

Based on the analysis above, this paper proposed the following research hypothesis (Figure 1):

**H1:**  *Both horizontal and vertical agricultural divisions of labor have positive impact on fertilizer reduction application*.

**H2:**  *Agricultural horizontal division of labor promotes fertilizer application reduction by enhancing internal specialization*.

**H3:**  *Agricultural vertical division of labor promotes fertilizer reduction by involving external socialized services*.

## 3. Data and Methods

### 3.1. Data Collection

The data of this study are collected from a microscopic questionnaire survey of farmers in the main rice-producing areas of Sichuan Province from July to October 2021. Sichuan is the only major grain-producing province and the main rice-producing province in western China and is also one of the most important grain-producing regions in China [49,50]. Rice is the number one grain crop in Sichuan’s agricultural production, and it also has a decisive position in China. According to the data from the National Bureau of Statistics of China, Sichuan Province applied 2.1082 million tons of fertilizer in 2020, which is a major province in fertilizer utilization and consumption.

**Figure 2:**
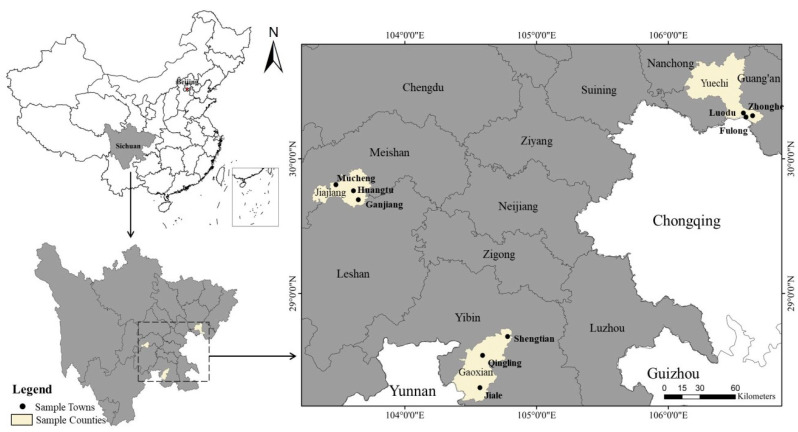
Distribution of sample villages.

This survey contains personal and household characteristics, land conditions, green production, technology adoption, and socialized services of the rice cultivators. The sampling steps were as follows (Figure 2) [51,52]: first, considering the level of economic development, we divided the 183 counties of Sichuan province into three groups, and we randomly selected one county from each group as a sample county and obtained three sample counties, including Jiajiang County, Yuechi County, and Gao County. Second, according to the difference of economic development and the distance from the center of the county government, we randomly divided the townships into three groups, and we selected one township from each group. According to similar sampling criteria, we selected three villages randomly from each township to obtain 27 sample villages; finally, we selected 20 households at random from each village to obtain a total of 540 valid sample data of farmers.

### 3.2. Variable Definitions

#### 3.2.1. Explained Variable

Fertilizer reduction application is the explained variable of this paper, which reflects whether the economic input behavior of farmers in the production process is optimal for fertilizer. On the basis of referring to Qiao and Huang [15], this paper chose the logarithmic Cobb–Douglas production function model to measure “Whether fertilizer is applied in reduction”. If the result is “Yes “, the value is 1, and “No” is 0.

#### 3.2.2. Kernel Explaining Variable

(1) Horizontal division of labor. Agricultural horizontal division of labor is expressed the degree of specialization in rice cultivation. Based on Liang et al. [26] and Ben Bradshaw [53], the degree of specialization in the agricultural division of labor was measured by the contiguity of rice cultivation area. This paper chose the Herfindahl-Hirschman Index (HHI) to measure [54]. Its mathematical expression is as follows:

(1) $$HHI=\sum_{i=1}^{n}{\left(\frac{S_{ij}}{X_{j}}\right)}^{2}$$

where, $S_{ij}$ is the total cultivated area of the *i*-th crop of farmer *j*, and $X_{j}$ is the total area of rice cultivated by farmer *j*. The HHI is a constant between 0 and 1; if its value is higher, it indicates a higher degree of specialization of farmers.

(2) Vertical division of labor. Agricultural vertical division of labor is expressed as the adoption of socialized services in the ante-production, production, and post-production stages, partially or completely [27], and this paper chose whether to purchase socialized services to measure vertical division of labor. If the result is “Yes “, the value is 1, and, if it is “No”, it is 0.

#### 3.2.3. Control Variable

To minimize the influence of omitted variables on agricultural division of labor on fertilizer reduction application, control variables were selected in this paper to further examine the influencing between variables. They are divided into the following three categories: ① individual characteristics, including gender, age, and education of household head [15]; ② household characteristics, including household labors [33] and agricultural income proportion [16]; and ③ land characteristics, including number of plots and average plot size [17], soil fertility [19], hydraulic conditions [55], terrain [56], and distance from the farmer’s home to the land [29]. In addition, this paper included regional dummy variables to control for regional differences. The definition of each variable is shown in Table 1.

### 3.3. Empirical Model

#### 3.3.1. Model of the Production Function to Measure Whether Farmers Apply Fertilizer Reduction or Not

In order to measure whether farmers adopt the economically optimal input behavior of fertilizer reduction, first, we establish a Cobb-Douglas (C-D) production function where the rice output income (yield) is used as the dependent variable and includes the fundamental elements of production required in rice cultivation activities, which include seed input (seed), machinery input (machine), fertilizer input (fertilizer), and pesticide input (pesticide) as the independent variables. We have constructed the following economic model:

(2) $$\ln\left(\mathrm{yield}\right)=\beta_{0}+\beta_{1}\ln\left(\mathrm{seed}\right)+\beta_{2}\ln\left(\mathrm{machine}\right)+\beta_{3}\ln\left(\mathrm{fertilizer}\right)+\beta_{4}\ln\left(\mathrm{pesticide}\right)+\varepsilon$$

where $\beta_{0}$ is a constant term, $\beta_{1}\ldots \ldots \beta_{i}$ are regression coefficients, and ε is a random disturbance term.

Here, $\beta_{3}$ is the fertilizer input elasticity and is based on the profit maximization theory, and the maximum benefit for farmers is that the marginal benefit is equal to the marginal cost, that is:

(3) $$\frac{\partial \mathrm{yield}}{\partial \mathrm{fertilizer}}=\frac{P\mathrm{fertilizer}}{P\mathrm{yield}}$$

We obtain the optimal fertilizer input for the farmer by the association of Equations (2) and (3):

(4) $$F_{optimal}=\frac{\beta_{3}\times \mathrm{yield}\times P\mathrm{fertilizer}}{P\mathrm{yield}}$$

Thus, we can judge whether the fertilizer application by farmers is excessive or not by comparing the marginal output to input ratio in rice production activities with 1:

(5) $$\frac{\beta_{3}\times \mathrm{yield}\times P\mathrm{fertilizer}}{\mathrm{fertilizer}\times P\mathrm{yield}}=\frac{\beta_{3}\times PF}{PY}=1$$

Here, if the marginal ratio is above 1, it indicates that the fertilizer application is economic; if the marginal ratio is less than 1, it indicates that the fertilizer application is not economic and has been excessive.

#### 3.3.2. Model of Factors Impacting Whether Farmers Apply Fertilizer Reduction or Not

For the dependent variables, “Whether fertilizer is applied in reduction” is a dichotomous variable. Therefore, we choose a binary probit model to explore the impact of the agricultural division of labor on whether farmers apply fertilizer reduction behavior. The function is the following:

(6) $$Y=ln(\frac{P_{i}}{1-P_{i}})=\alpha_{0}+\beta_{1}X_{1}+\beta_{2}X_{2}+\ldots \ldots +\beta_{n}X_{i}+\varepsilon$$

where, $P_{i}$ is the probability of farmers’ fertilizer reduction behavior; $\alpha_{0}$ is a constant term, $X_{1}\ldots X_{i}$ are independent variables, including kernel explaining variables, control variables and regional dummy variables, $\beta_{1}\ldots \ldots \beta_{i}$ are regression coefficients, and ε is a random disturbance term.

## 4. Results

This section may be divided by subheadings. It should provide a concise and precise description of the experimental results, their interpretation, as well as the experimental conclusions that can be drawn.

### 4.1. Measurement of Whether Farmers Apply Fertilizer Reduction or Not

The results of the C–D production function in Table 2 indicate that fertilizer has a significant positive effect on rice production, which is significant at a 1% level and its production elasticity is 0.375. Specifically, with optimal fertilizer application, fertilizer input increased by 1%, and rice production increased by 0.375%. Furthermore, seed, machinery, and pesticide inputs all have significant positive effects on rice output.

### 4.2. The Effect of Agricultural Division of Labor on Whether Farmers Apply Fertilizer Reduction or Not

Table 3 shows the estimation results of the agricultural division of labor on whether fertilizer reduction is applied by farmers. Here, model (1) and model (2) show the results of estimating the Porbit model controlling for regional dummy variables. For purpose of interpreting these coefficients, the results in Table 3 show the marginal effects of the models.

Both horizontal and vertical agricultural division of labor have positive and significant effects on the reduction in fertilizer application by rice farmers, which are significant at the 5% and 1% levels, respectively. Specifically, all other conditions being equal, the horizontal division of labor and vertical division of labor increased by 17.2% and 19.1% units of fertilizer reduction, respectively, for each unit increase. It is consistent with the empirical evidence of Liang et al. [29]. This indicates that, with the deepening of horizontal and vertical division of labor, farmers will tend to more often reduce the application of fertilizer. Thus, it supports the central hypothesis H1 presented above.

As for the household characteristics, where both labor and agricultural income rate are significant at the 1% level, this indicates that the adjustments in labor structure and income structure have a significant negative effect on fertilizer reduction, and it is possible that the unscientific structure of production elements, the more fertilizer application by farmers may be as well, which is same as the conclusions from Lu and Xie [16]. A possible explanation is that households with more labor, which may have higher economic pressure and higher risk aversion, tend to apply more fertilizer to avoid yield losses [33], while farmers with a higher proportion of agricultural income trust their production methods will lead to high returns rather than decreasing their conventional fertilizer application [42]. Therefore, it is necessary to adjust the structure of traditional elements inputs. It confirms the previous hypothesis, H2. As for land characteristics, both the number of non-square plots and average plot size are significant at the 1% level, which indicates improvement in land elements has a significant effect on fertilizer reduction. The higher number of non-square plots and average plot size can effectively reduce the application of fertilizer, which is the same as the results obtained by Wu et al. [30]. Hu, Y. and Zhang, Z. [57] also confirmed that land characteristics significantly affect farmers’ adoption of outsourced services, but they do not directly affect fertilizer inputs, but they indirectly affect farmers’ fertilizer inputs through the availability and price of socialized agricultural services. Thus, this confirms hypothesis H3. Furthermore, the effect of individual characteristics of farmers on fertilizer reduction is not significant, which indicates that fertilizer application is mainly a habitual and fixed behavior based on experience, whereas the effect of individuals is not significant [38].

### 4.3. Robustness Test

#### Measurement of Kernel Explaining Variable

This paper tests the robustness on the basis of the original model, which is shown in Table 4. One is to change the regression model, changing the probit model used above to the logit model. Model (1) and model (2) show the estimation results of the horizontal division of labor and vertical division of labor. The comparison of the regression results shows that the alternative model is stable in trends to the original model with only minor differences in the coefficients. It indicates that the results are robust; another is to use alternative kernel explaining variables. As for the horizontal division of labor, we use willingness to pay for socialized services as a substitute variable for the vertical division of labor. It is likely that the stronger the willingness to pay for socialized services, the increased the recognition and acceptance of fertilizer technology by farmers will be, and thus the adoption of green production to reduce fertilizer application will be easier [58]. Model (3) regression results show that the willingness to purchase socialized services has a significant effect on fertilizer reduction, which is significant at the 1% level, consistent with the previous results. As for the vertical division of labor, referring to Liang et al. [29], we use the cost of socialized services per mu of farmers to substitute the horizontal division of labor, which reflects both whether farmers participate in the vertical division of labor and the degree of their involvement. The extent to which farmers are willing to pay reflects the level of demand for socialized services, which will influence the way they divide agricultural labor, leading to agricultural fertilizer reduction [59]. The regression results in the model (4) show that the cost of socialized services per mu has a significant positive effect on fertilizer reduction, which also confirms the previous conclusions. At the same time, the above test further reconfirms that the deeper the involvement of farmers in the agricultural horizontal and vertical division of labor, the more possible the fertilizer reduction.

Furthermore, to overcome the possible bias of the model, referring to Zhang et al. [34] and Liang et al. [29] considering that topography can have an impact on agricultural production and that topography is often considered as an instrumental variable for green production socialization services, this paper uses topography as a criterion for grouping on the basis of the original regression. Table 5 shows the results of the effect of the agricultural division of labor on fertilizer reduction in slope and plain terrain groups, respectively. The results show that the agricultural division of labor has a positive and significant effect on fertilizer reduction, which is consistent with the previous analysis, indicating robustness. Additionally, we notice that the horizontal division of labor of slope is more significant than plain for fertilizer reduction, while the vertical division of labor of plain is more significant than slope, which further expanded the conclusions obtained above. This may be due to the relatively developed level of socio-economic development in the plain areas, which is more conducive to the promotion of mechanization and the deepening of vertical division of labor with the help of external socialized services, while the sloping areas tend to adopt horizontal division of labor due to the constraints and make up for the gap through internal labor restructuring.

### 4.4. Endogeneity Test

Due to the problems of measurement error, omitted variables, and reverse causality, this may result in endogeneity of models. It is an effective solution to relieve the endogeneity problem by finding appropriate instrumental variables for the kernel explaining variables. Considering fertilizer reduction application, which is a binary variable, but not a continuous variable, this paper refers to Zou et al. [60], who proposed the conditional mixed process (CMP) method, which is used to deal with the potential endogeneity problem. Additionally, the estimation result of CMP is better than the IV-Probit model, which only works for continuous variables.

Besides, the instrumental variables are required to be significantly associated with endogenous variables (horizontal division of labor and vertical division of labor) while not directly impacting the explained variable (fertilizer reduction application). In this paper, we use the cost of machinery services as an instrumental variable of horizontal division of labor. On the one hand, the adoption of machinery services is based on continuous and large-scale production, and the specialized machinery services match with the scale land [17]. On the other hand, the value of specialized service cost reflects the depth of specialization, but the cost itself does not directly affect fertilizer reduction. Similarly, we use terrain as an instrumental variable for the vertical division of labor. The limitation of terrain conditions significantly affects the outsourcing of socialized services in rice production, which is a labor-intensive production activity [61], but terrain does not directly affect fertilizer reduction. Rather, it plays a role through the influence of indirect elements, such as the availability, cost, and price of agricultural socialized services.

Table 6 shows the results of the CMP method. atanhrho_12 represents the residual correlation of the two-stage regression model of CMP, and its coefficients are significant at the 1% level, as well as not 0. It indicates that the model is endogenous, and our test is necessary. The first stage estimation results of model (1) and model (3) show that the instrumental variables are highly correlated with the endogenous explained variables, which are significant at the 1% and 10% levels, respectively. Thus, it satisfies the hypothesis on the correlation for instrumental variables. Therefore, our selected instrumental variables are valid, and the estimation results are reliable. Model (2) and model (4) show that both horizontal and vertical agricultural divisions of labor have positive and significant effects on the reduction in fertilizer application. Therein, both horizontal and vertical division of labor are significant at the 1% level, whose coefficients were 2.659 and 2.036. These empirical results are consistent with the previous conclusions.

## 5. Mechanism Analysis

Within the resource constraint of agricultural productivity and production structure adjustment, which is based on the advantage of resource endowment, we consider the optimal allocations of elements that can accurately explore the degree of fertilizer reduction application [53]. Thus, in order to further examine the conclusion above, we analyze the previous origin regression model. First, we interact horizontal division of the number of household labors with the number of labor and the agricultural income radio under the constraints of factor structure and land characteristics to explore the effects of internal specialization in the horizontal division of the labor and the structure of elements on fertilizer reduction. Then, we interact vertical division of labor with the number of plots and hydraulic conditions to explore the effects of external socialized services and land characteristics on fertilizer reduction. The results are shown in Table 7.

The coefficients of the horizontal division of labor are all positive and significant at the 1% level. Agricultural horizontal division of labor promotes fertilizer application reduction by enhancing internal specialization. It suggests that the horizontal division of labor of farmers and the adjustment of elements’ structure can promote specialization in the horizontal division of labor. To achieve economies of scale, farmers increase specialization in production, resulting in reducing marginal cost and application of fertilizer. It contributes to achieving labor and agricultural capital accumulation, which improves the fertilizer application efficiency, resulting in reducing marginal cost so that farmers prefer made chemical fertilizer reductions. The coefficients of the vertical division of labor are positive and significant at the 1% level and 10% level, respectively. This explains that agricultural vertical division of labor promotes fertilizer reduction by involving external socialized services. Additionally, it shows that new technologies and efficient production processes are related to the fixed land resource endowment with the adoption of social services. Thus, it is possible to improve the actual fertilizer utilization rate and facilitate fertilizer reduction by farmers. Similarly, the results of the model estimation support the conclusions shown above.

## 6. Discussion

This paper applies the theory of division of labor to the field of agriculture, establishes an innovative theoretical framework for analyzing the influence of agricultural division of labor on fertilizer reduction application, and creatively proposes how rice farmers should make reasonable decisions on horizontal and vertical division of labor and make optimal fertilizer application behavior. This paper found that both horizontal and vertical division of labor in agriculture could significantly promote fertilizer reduction application by rice farmers, which is the same as the conclusion of Liang et al. [29]. Specialized management and socialized service can cope well with the transfer of labor force and the development of modern agriculture. In order to achieve economies of scale, farmers improve their specialization, adopt socialized outsourcing services, and reduce the amount of fertilizer application through scientific, professional, and unified guidance, which is the same as the conclusion of Zhang et al. [27]. However, it is contrary to the conclusion of Lu and Xie [16]. They found that production specialization reduces agricultural labor resources and induces farmers to use more fertilizer in order to increase output, which is related to farmers’ ability to bear risks [15,33].

Although some practical conclusions are obtained in this paper, there are also some limitations, which need to be further explored and improved in future studies: (1) The indicators of agricultural division of labor are relatively simple. In the theory of agricultural division of labor, we selected the most representative degree of specialization and socialized service, respectively, to measure the impact on farmers’ fertilizer reduction. There may be other indicators not included in the analysis. Future studies can try to analyze from a more comprehensive level. (2) The dynamic nature of environmental sustainability is not considered. Due to time, funding, and data collection limitations, this study is based only on survey data from 2020 and lacks long-term studies and comparisons. However, in future studies, we can try to make further comparative analysis from a dynamic perspective by using survey tracking data.

## 7. Conclusions and Policy Implications

Based on 540 survey data of farmers in the main rice-producing areas of Sichuan Province, this paper empirically analyzes the impact of the agricultural division of labor on fertilizer reduction application. The results show that both horizontal and vertical agricultural divisions of labor have positive and significant effects on the reduction in fertilizer application by rice farmers. After changing the model and replacing the core variables and grouping, the results remain stable. With consideration of the impact of the potential endogenous problems, the positive effect remains significant by using the CMP method. We make further mechanical analysis and found that, due to the migration of rural labor, the horizontal division of labor is expressed in changes in the structure of labor and cultivation within the household, which has changed. Farmers increase specialization in production, resulting in reduced fertilizer. Similarly, the vertical division of labor is expressed in the adoption of external socialized services by farmers, which improves the land resource endowment, which in turn promotes fertilizer reduction by farmers.

Fertilizer reduction as the key work to achieve sustainable agricultural development, as well as how to better promote farmers’ fertilizer reduction behavior, are currently the main problems in agricultural production. Based on the analysis and conclusions above, this paper gives the following policy implications:

First, the government should motivate farmers to deepen their participation in the horizontal and vertical division of labor. For example, consider the application of horizontal agricultural division of labor: the government promotes organizational forms, such as cooperatives, family farms, and farmers’ professional cooperatives, to promote division of labor and cooperation among farmers, form large-scale production, and optimize product structure, thus reducing the application of chemical fertilizers. It also gives tax incentives, subsidies, and other policies to encourage farmers to actively participate in organized production. Application of vertical agricultural division of labor is also considered. The government strengthens technical guidance to farmers and promotes green production models by introducing agricultural science and technology achievements and services externally, organizing technical training, and providing technical consultation.

Second, it is continuously improving the level of agricultural specialization and adjusting the structure of the labor force and planting structure. Therefore, it is necessary to further make a better environment for migrant workers who seek employment, reducing the risk of non-farm employment and guaranteeing the stability of the income they earn. In addition, it is necessary to arrange the structure of agricultural planting in a rational way to play the superiority of specialized production, which reduces the production transaction cost. It is also needed to improve the income of farmers so as to increase their ability to afford the green production technology in order to achieve a reduction in chemical fertilizer.

Third, it is supposed to pay attention to further promote the market of socialized agricultural services. On the one hand, we should encourage farmers to outsource services in all aspects of production and expand the market demand for outsourcing agricultural machinery services. Additionally, the government should develop the agricultural socialized service system, cultivate and strengthen the number of companies and businesses providing specialized agricultural services in markets, guarantee service supply and standardize quality, and promote the deepening of agricultural vertical division of labor. On the other hand, the government should reinforce the technological research and extension of fertilizer application, as well as actively promote the application of green technologies, such as organic fertilizer and formulated fertilization and straw returning. Additionally, it plays the role of guidance and education to farmers. By increasing the substitution of new factors for traditional elements to change the original unscientific production methods, we can enable farmers to reduce the application of chemical fertilizers in their production.

## Figures and Tables

**Table 1:** Variable definition and descriptive statistics.

Variables	Observed Variables	Descriptions	Mean	S.D.
Input–output variable	Rice output	Rice output income (Yuan/Mu)	899.00	579.80
	Seed input	Seed input costs (Yuan/Mu)	58.28	53.92
	Machine input	Machine input costs (Yuan/Mu)	76.27	107.60
	Fertilizer input	Fertilizer input costs (Yuan/Mu)	118.80	148.60
	Pesticide input	Pesticide input costs (Yuan/Mu)	50.33	83.01
Explained variable	Fertilizer reduction application	Whether fertilizer is applied in reduction (1 = Yes; 0 = No)	0.49	0.50
Kernel explaining variable	Horizontal division of labor	Herfindahl-Hirschman Index, HHI	0.51	0.40
	Vertical division of labor	Whether the purchase of socialized services (1 = Yes; 0 = No)	0.69	0.46
Individual characteristics	Gender	Gender of household head (1 = Male; 0 = Female)	0.11	0.31
	Age	Age of household head (Year)	58.93	11.02
	Education	Years of education of household head (Year)	6.75	3.17
Household characteristics	Labor	The number of household labors (Person)	2.63	1.45
	Rice income proportion	Rice income in proportion to total income (%)	41.37	33.14
	Agricultural income proportion	Agricultural income in proportion to total income (%)	23.49	28.36
Lands characteristics	Number of plots	The number of plots being operated (Piece)	14.40	46.76
	Number of non-square plots	The number of non-square plots being operated (Piece)	11.03	43.36
	Average plot size	Average plot size (Mu)	0.46	0.60
	Soil fertility	Soil fertility (1 = Very bad; 2 = Bad; 3 = General; 4 = Good; 5 = Very good)	3.05	1.07
	Hydraulic conditions	Hydraulic infrastructure conditions (1 = Very bad; 2 = Bad; 3 = General; 4 = Good; 5 = Very good)	3.14	1.40
	Water and soil erosion	Degree of water and soil erosion (1 = Very bad; 2 = Bad; 3 = General; 4 = Good; 5 = Very good)	2.42	1.18
	Distance	Distance from the farmer’s home to the nearest land being operated (Meter)	210.20	362.70

**Table 2:** Result of regression of C–D production function (log).

Variable	Rice
Seed	0.923 ***
	(0.045)
Machine	0.112 ***
	(0.016)
Fertilizer	0.375 ***
	(0.036)
Pesticide	0.156 ***
	(0.039)
Constant	0.435 ***
	(0.077)
r^2^	0.913
N	540

Note: * *p* < 0.1, ** *p* < 0.05, *** *p* < 0.01.

**Table 3:** Regression results of the effect of agricultural division of labor on fertilizer reduction application.

Variable	(1)	(2)
Horizontal division of labor	0.172 **	
	(0.082)	
Vertical division of labor		0.191 ***
		(0.045)
Gender	−0.042	−0.042
	(0.067)	(0.066)
Age	0.004 **	0.004 *
	(0.002)	(0.002)
Education	0.005	0.005
	(0.007)	(0.007)
Labor	−0.009 ***	−0.010 ***
	(0.003)	(0.003)
Rice income rate	0.002	0.002
	(0.002)	(0.003)
Agricultural income rate	−0.155 ***	−0.161 ***
	(0.030)	(0.030)
Number of plots	−0.004	−0.010
	(0.016)	(0.016)
Number of non-square plots	0.004 ***	0.004 ***
	(0.001)	(0.001)
Average plot size	0.002 ***	0.002 **
	(0.001)	(0.001)
Soil fertility	0.004	0.002
	(0.021)	(0.020)
Hydraulic conditions	0.002	0.010
	(0.016)	(0.016)
Water and soil erosion	0.021	0.018
	(0.019)	(0.018)
Distance	−0.000	−0.000
	(0.000)	(0.000)
Regional dummies	Yes	Yes
r^2^	0.1337	0.1492
N	540	540

Note: * *p* < 0.1, ** *p* < 0.05, *** *p* < 0.01.

**Table 4:** Regression results based on the changing model and substituting kernel explaining variables.

Variable	(1)	(2)	(3)	(4)
Horizontal division of labor	0.172 **			
	(0.081)			
Vertical division of labor		0.191 ***		
		(0.045)		
Willingness to pay for socialized services			0.185 ***	
			(0.047)	
Cost of socialized services per mu				0.004 ***
				(0.001)
Control	Yes	Yes	Yes	Yes
Regional dummies	Yes	Yes	Yes	Yes
r^2^	0.1333	0.1492	0.1465	0.1667
N	540	540	540	540

Note: * *p* < 0.1, ** *p* < 0.05, *** *p* < 0.01.

**Table 5:** Regression results based on grouping in terms of terrain.

Variable	Slope		Plain	
Horizontal division of labor	0.733 **		0.236	
	(0.331)		(0.263)	
Vertical division of labor		0.446 *		0.635 ***
		(0.235)		(0.191)
Control	Yes	Yes	Yes	Yes
Regional dummies	Yes	Yes	Yes	Yes
r^2^	0.1742	0.1703	0.1673	0.1944
N	257	257	283	283

Note: * *p* < 0.1, ** *p* < 0.05, *** *p* < 0.01.

**Table 6:** Result of CMP method.

Variable	(1)	(2)	(3)	(4)
Horizontal division of labor		2.659 ***		
		(0.332)		
Vertical division of labor				2.036 ***
				(0.674)
Cost of machinery services	0.036 ***			
	(0.006)			
terrain			0.093 *	
			(0.048)	
Control	Yes	Yes	Yes	Yes
Regional dummies	Yes	Yes	Yes	Yes
lnsig_2	−1.248 ***		−0.886 ***	
	(0.030)		(0.030)	
atanhrho_12	−0.911 ***		−0.807 *	
	(0.205)		(0.628)	
N	540	540	540	540

Note: * *p* < 0.1, ** *p* < 0.05, *** *p* < 0.01.

**Table 7:** Moderating effects of the agricultural division of labor on fertilizer application reduction.

Variable	(1)	(2)	(3)	(4)
Horizontal division of labor	0.345 **	0.358 ***		
	(0.143)	(0.099)		
Vertical division of labor			0.290 ***	0.234 *
			(0.064)	(0.119)
Interaction between horizontal division of labor and the number of household labors	−0.063			
	(0.041)			
Interaction between horizontal division of labor and agricultural income radio		0.006 ***		
		(0.001)		
Interaction between vertical division of labor and the number of land plots			−0.008 **	
			(0.004)	
Interaction between vertical division of labor and hydraulic conditions				−0.013
				(0.033)
Control	Yes	Yes	Yes	Yes
Regional dummies	Yes	Yes	Yes	Yes
r^2^	0.1296	0.1337	0.1407	0.1492
N	540	540	540	540

Note: * *p* < 0.1, ** *p* < 0.05, *** *p* < 0.01.

## Data Availability

We can provide raw data and code if needed.
